# Quantitative profiling of sphingolipids in wild Cordyceps and its mycelia by using UHPLC-MS

**DOI:** 10.1038/srep20870

**Published:** 2016-02-12

**Authors:** Jia-Ning Mi, Jing-Rong Wang, Zhi-Hong Jiang

**Affiliations:** 1 State Key Laboratory of Quality Research in Chinese Medicine, Macau Institute for Applied Research in Medicine and Health, Macau University of Science and Technology, Macau, China; 2 International Institute for Translational Chinese Medicine, Guangzhou University of Chinese Medicine, Guangzhou, China

## Abstract

In the present study, 101 sphingolipids in wild Cordyceps and its five mycelia were quantitatively profiled by using a fully validated UHPLC-MS method. The results revealed that a general rank order for the abundance of different classes of sphingolipids in wild Cordyceps and its mycelia is sphingoid bases/ceramides > phosphosphingolipids > glycosphingolipids. However, remarkable sphingolipid differences between wild Cordyceps and its mycelia were observed. One is that sphingoid base is the dominant sphingolipid in wild Cordyceps, whereas ceramide is the major sphingolipid in mycelia. Another difference is that the abundance of sphingomyelins in wild Cordyceps is almost 10-folds higher than those in most mycelia. The third one is that mycelia contain more inositol phosphorylceramides and glycosphingolipids than wild Cordyceps. Multivariate analysis was further employed to visualize the difference among wild Cordyceps and different mycelia, leading to the identification of respective sphingolipids as potential chemical markers for the differentiation of wild Cordyceps and its related mycelia. This study represents the first report on the quantitative profiling of sphingolipids in wild Cordyceps and its related mycelia, which provided comprehensive chemical evidence for the quality control and rational utilization of wild Cordyceps and its mycelia.

Cordyceps is a composite consisting of the stroma of the fungus *Cordyceps sinensis* [Berk.] Sacc. (family Hypocreaceae) and the dead caterpillar of *Hepialus armoricanus* (family Hepialidae) whose larva is the primary host of the fungus. Cordyceps is one of the most famous and expensive traditional Chinese medicine and healthy food. It is restrictedly distributed in alpine habitats on the Tibetan Plateau in China. Since Cordyceps has the actions of tonifying kidney, replenishing lung, stanching bleeding and resolving phlegm, it has been historically used in clinic for the treatment of hyposexualities, hyperglycemia, hyperlipidemia, asthemia, respiratory disease, and renal dysfunction, etc.^1^,^2^. In addition to the medicinal applications, Cordyceps is also widely utilized as a tonic and functional food in China.

The broad spectrum of pharmacological effects of wild Cordyceps has led to an ever-increasing demand on this herbal medicine whose natural resource is increasingly scarce. To meet the consumption demands of wild Cordyceps, several mycelia products have been developed and manufactured in large quantities by using fermentation technology. To date, five mycelia of different fungus isolated from wild Cordyceps have been approved as drugs by China Food and Drug Administration (CFDA), including *Cordyceps sinensis*, *Hirsutella sinensis*, *Cephalospovium sinensis*, *Mortierella SP* and *Gliocadium roseum*. According to the data from National Bureau of Statistics of China, the annual production of these mycelia was over 3000 tons in 2011, demonstrating wide application and recognized therapeutic effects of these mycelia products.

As alternatives of wild Cordyceps, mycelia have been substantially investigated for their pharmacological effects, showing that the mycelia could invigorate the lung and nourish kidney, improve the actions of heart and liver, and increase immunity, etc.^3^,^4^,^5^,^6^,^7^,^8^,^9^,^10^. However, comprehensive studies on chemical constituents of individual mycelium are still absent. Currently, nucleosides, mannitol and amino acids which are not specific constituents of either mycelia or wild Cordyceps were used as markers for the quality control of these mycelia. In addition, reported bioactivities of above constituents are not fully responsible for the observed effects of the mycelia. These limitations highlight the importance of exploring specific and active constituents of mycelia for quality control.

In 1994, myriocin, a natural sphingolipid (SPL), was isolated from the culture broth of *Isaria sinclairii* (the imperfect stage of *Cordyceps sinclairii*) as a potent immunosuppressive constituent^11^. Starting from myriocin, FTY720 was synthesized and finally developed into a drug (Fingolimod) for the treatment of multiple sclerosis and was studied in phase III clinical trial of organ transplantation. This implies that SPLs might be active constituents of wild Cordyceps and its mycelia.

SPLs are a complex family of compounds that share a common structural feature, a sphingoid base backbone which is biosynthesized from serine and a long-chain fatty acyl-CoA, and then converted into ceramides, phosphosphingolipids, glycosphingolipids and other species^12^. Substantial studies have demonstrated crucial role of endogenous SPLs in various biological procedures. Meanwhile, numerous studies have revealed significant bioactivities of natural and chemically synthesized SPLs. For instance, sphingoid bases have been regarded as potential anticancer agents, as represented by safingol^13^,^14^ and 1-deoxysphinganine^15^,^16^, both of which are being evaluated in phase I clinical trials. Recent study also suggested that structural analogues of ceramide (C16-serinol) and exogenous natural ceramide exhibited promising anticancer effects^17^,^18^. Additionally, evidence showed that sphingomyelin has effects on the post-initiation development of preneoplastic lesions in the rat colon^19^. These evidence strongly suggested that natural SPLs are pharmacologically active constituents of natural medicines. Therefore, comprehensive study on the SPLs in wild Cordyceps and its mycelia is desperately needed for the clarification of substantial basis of their pharmacological activities and therapeutic effects. We herein carried out a quantitative profiling of the SPLs of wild Cordyceps and its five mycelia by using an improved sphingolipidomic approach that has been established in our lab^20^.

## Results

### Identification of SPLs in Cordyceps mycelia

Using the method established in our lab^20^, SPLs in samples were identified on the basis of high-resolution MS and MS/MS data, matching of SPLs with comprehensive SPL database, and confirmation of SPL standards. As the result, a total of 101 SPLs were identified from a pooled sample of five mycelia (Supplementary material, Table S1), including 10 sphingosines (So), 6 sphinganines (Sa), one sphingoid base 1-phosphate (S1P), one lysosphingomyelin (S1Po), 51 ceramides (Cer), 8 hexosyl ceramides (HexCer), 19 sphingomyelins (SM), 3 inositol phosphorylceramides (PI-Cer) and 2 mannosylinositol phosphorylceramides (MIPC). It can be seen that Cers are the most diverse SPLs in mycelia, followed by SMs and Sos. Among the 101 identified SPLs, 88 species were also identified in wild Cordyceps.

### Method validation for quantitation

To quantitatively profile the SPLs, a MRM-based method established in our lab was further validated for the quantification of SPLs in wild Cordyceps and its mycelia.

#### Linearity, limit of detection (LOD) and limit of quantitation (LOQ)

As shown in Table 1, all 6 calibration curves exhibited good linearity (correlation coefficients r^2^ ≥ 0.9977) over wide dynamic ranges which spanned more than 2 orders of magnitude. The LOD ranged between 0.005 and 3.34 nM, whereas LOQ ranged between 0.0167 and 16.7 nM.

#### Injection precision, intra- and inter-day precision

As shown in Table 2, the relative standard deviations (RSD) of the levels of all quantified endogenous SPLs in samples were less than 6% for injection precision tests. Satisfactory RSD median values (3.79% and 3.96%) were achieved for intra- and inter-day precisions.

#### Stability

As shown in Table 2, The RSDs for the levels of approximately 90% endogenous SPLs in samples were less than 10% for stability tests. The RSDs for the levels of over 90% endogenous SPLs in QC samples were less than 15% across the whole process.

#### Recovery

As shown in Table 1, mean recovery of 87.05%, 83.72%, 81.70%, 100.15%, 99.42% and 88.43% were achieved, respectively, for internal standards (IS) So (d17:1), S1P (d17:1), Sa (d17:0), Cer (18:1/12:0), GlcCer (d18:1/12:0) and SM (d18:1/12:0). RSD values of the recoveries of all IS at all spiking levels were less than 5%.

#### Accuracy

As shown in Figure S1 (Supplementary material), a good correlation between the added and determined amounts of individual SPLs with different carbon chains was examined. The fitting equation slopes were between 0.95 and 1.08 and correlation coefficients r^2^ ≥ 0.991.

### Quantitative comparison of SPLs in wild Cordyceps and its mycelia

**Figure 1:**
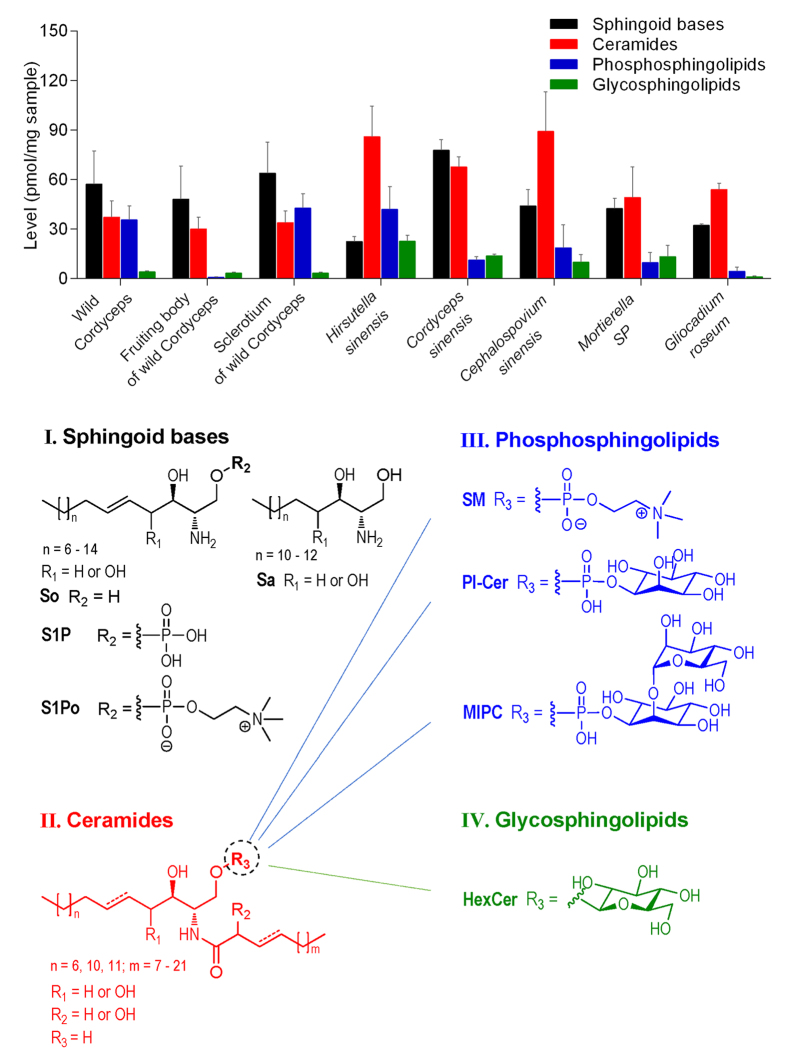
The total levels of four classes of SPLs in wild Cordyceps and its mycelia (mean ± SD of the total level of each class of SPLs).

By using the validated method, totally 101 SPLs in wild Cordyceps and its mycelia were quantitatively profiled. To view the overall distribution of SPLs, the total contents of four classes of SPLs were calculated (Fig. 1), which showed a rank order of sphingoid bases > ceramides > phosphosphingolipids ≫ glycosphingolipids in wild Cordyceps, and a different order (ceramides > sphingoid bases ≫ phosphosphingolipids/glycosphingolipids) in most mycelia. In mycelia, some special distribution features were observed for *Cordyceps sinensis* and *Hirsutella sinensis*. In *Cordyceps sinensis*, sphingoid bases were relatively rich and their total content was higher than that of ceramides, which is a reversed rank order of sphingoid bases and ceramides for the other mycelia. In *Hirsutella sinensis*, however, the total content of sphingoid bases was notably low whereas the abundance of phosphosphingolipids was relatively high, which led to a reversed rank order of sphingoid bases and phosphosphingolipids for the other mycelia.

The quantitative results also revealed distribution pattern of individual SPLs of wild Cordyceps and its mycelia, which was described by classes as below.

#### Quantification of sphingoid bases

**Figure 2:**
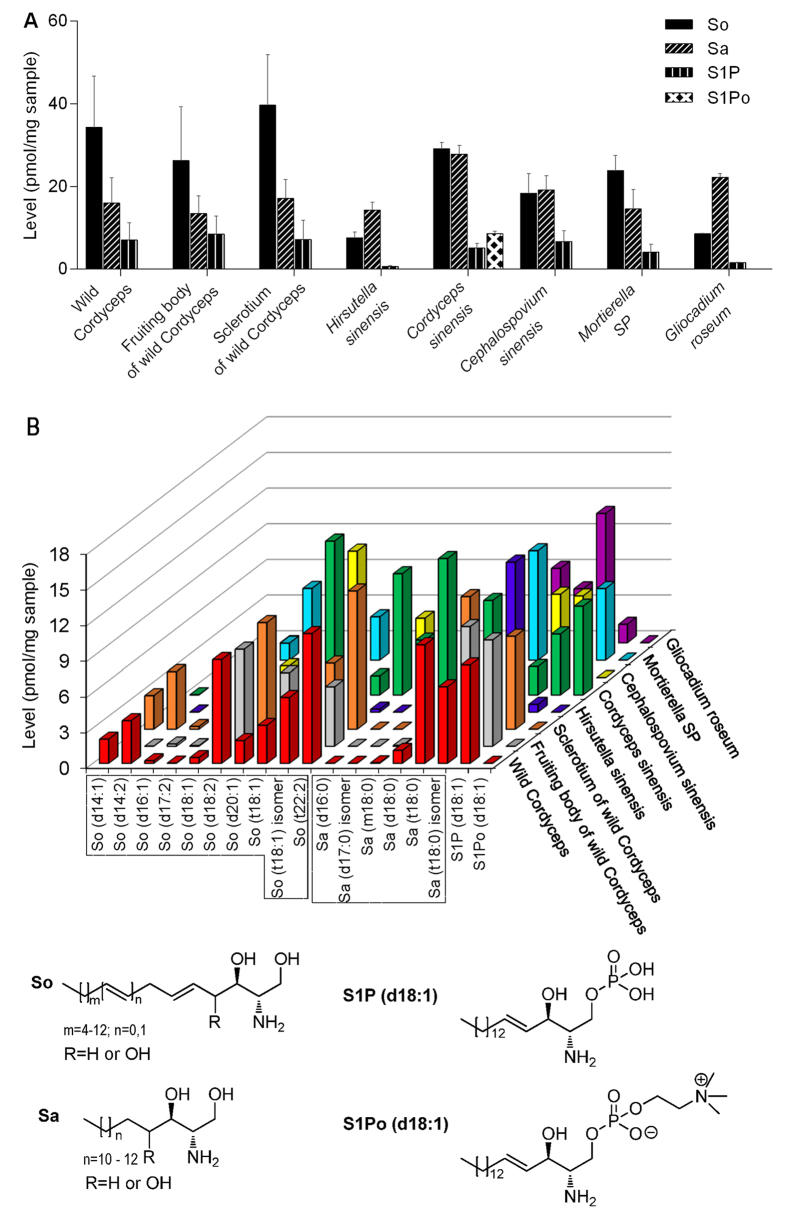
The total levels of four subclasses of sphingoid bases (**A**) each bar represents mean ± SD) and the levels of 18 sphingoid bases (**B**) each bar represents mean value of individual sphingoid bases) in wild Cordyceps and its mycelia.

Totally 18 sphingoid bases were quantified, including 10 Sos, 6 Sas, one S1P and one S1Po (Fig. 2). Levels of the four subclasses in both wild Cordyceps and its mycelia were generally in an order of So > Sa > S1P > S1Po, except for *Hirsutella sinensis* and *Gliocadium roseum*, in which Sa was more abundant than So and the content of S1P was extremely low (as low as 0.66 and 1.56 pmol/mg, vs about 7.17 pmol/mg in other samples). Structurally, sphingoid bases with carbon chain length >= 18 were more abundant than those species with carbon chain length <18 in terms of both number of species and content in all samples. When comparing different samples, the total content of sphingoid bases was found to be highest in *Cordyceps sinensis*, followed by wild Cordyceps (including individual parts fruiting body of wild Cordyceps and sclerotium of wild Cordyceps), *Cephalospovium sinensis*, *Mortierella SP*, *Gliocadium roseum* and *Hirsutella sinensis*. Of note, the total content of sphingoid bases in *Cordyceps sinensis* was more than 3-folds of that in *Hirsutella sinensis*.

#### Quantification of ceramides

Ceramides were the most structurally diverse species. A total of 51 ceramides were quantitatively determined (Figure S2). It can be seen that ceramides with d18 sphingoid base backbone (18 carbons of sphingoid base backbone with two hydroxyl groups) were the most structurally diverse (28–38 species) and dominant species, followed by those species with t18 sphingoid base backbone (18 carbons of sphingoid base backbone with three hydroxyl groups, also named phytosphingosine backbone) (9–10 species, with content of approximately 30% of that of d18 species). Of note, two uncommon Cers with odd-numbered sphingoid base backbone [Cer (d19:2/16:0) and Cer (d19:2/16:0(OH))] and one Cer with very short chain length sphingoid base backbone (d14) were found as low-abundance species in most samples. The d19 species was relatively rich in *Hirsutella sinensis* and wild Cordyceps. Comparison of the total content of Cers among samples suggested that *Cephalospovium sinensis* has the highest content of Cers, followed by other mycelia, whereas wild Cordyceps has the lowest content of Cers.

#### Quantification of phosphosphingolipids

**Figure 3:**
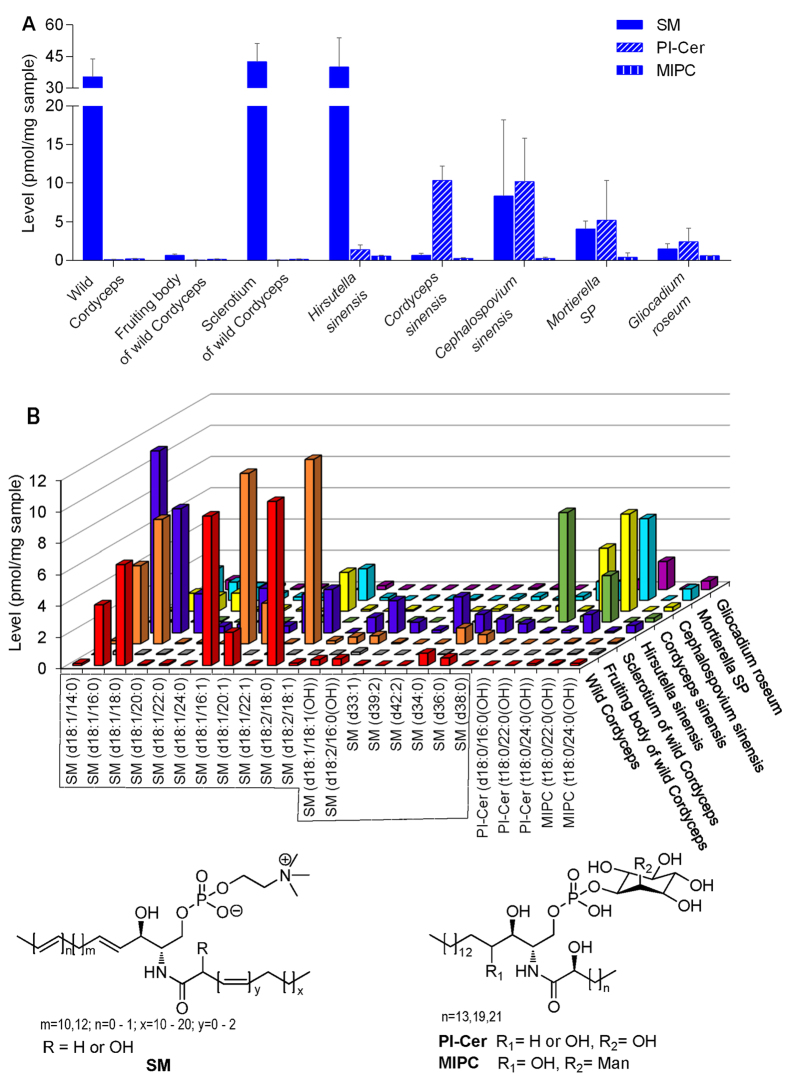
The total levels of three subclasses of phosphosphingolipids (**A**) each bar represents mean ± SD) and the levels of 24 phosphosphingolipids (**B**) each bar represents mean value of individual phosphosphingolipids) in wild Cordyceps and its mycelia.

As shown in Fig. 3, a total of 24 phosphosphingolipids belonging to three subclasses were measured, including 19 SMs, 3 PI-Cers and 2 MIPCs. In both wild Cordyceps and its mycelia, SMs and/or PI-Cers were dominant species whereas MIPCs were minor SPLs. Even though, the levels of SMs and PI-Cers varied remarkably. In *Cordyceps sinensis*, the abundance of SMs (0.69 pmol/mg) was about 14-folds lower than that of PI-Cers (10.32 pmol/mg), while in *Cephalospovium sinensis*, *Gliocadium roseum* and *Mortierella SP*, the total content of SMs (about 1.5–8.3 pmol/mg) was comparable to that of PI-Cers (2.4–10.2 pmol/mg). However, different from both *Cordyceps sinensis* and *Cephalospovium sinensis* etc, the level of SMs in *Hirsutella sinensis* and wild Cordyceps was notably high (about 35–42 pmol/mg), while PI-Cers was much less (about 0.1–1.4 pmol/mg).

#### Quantitation of glycosphingolipids

Glycosphingolipids observed in all samples belong to HexCers. Among which, species with sphingoid base bone of d19:2 accounted for majority of the structures (more than 80%). Totally 8 HexCers were quantified and the results were shown in Figure S3. It can be seen that in all samples, the total levels of HexCers in mycelia were generally 2–5 folds of that in wild Cordyceps, except for *Gliocadium roseum* of which the HexCer’s abundance was even lower than wild Cordyceps.

### Difference among sphingolipidomes of wild Cordyceps and its mycelia

**Figure 4:**
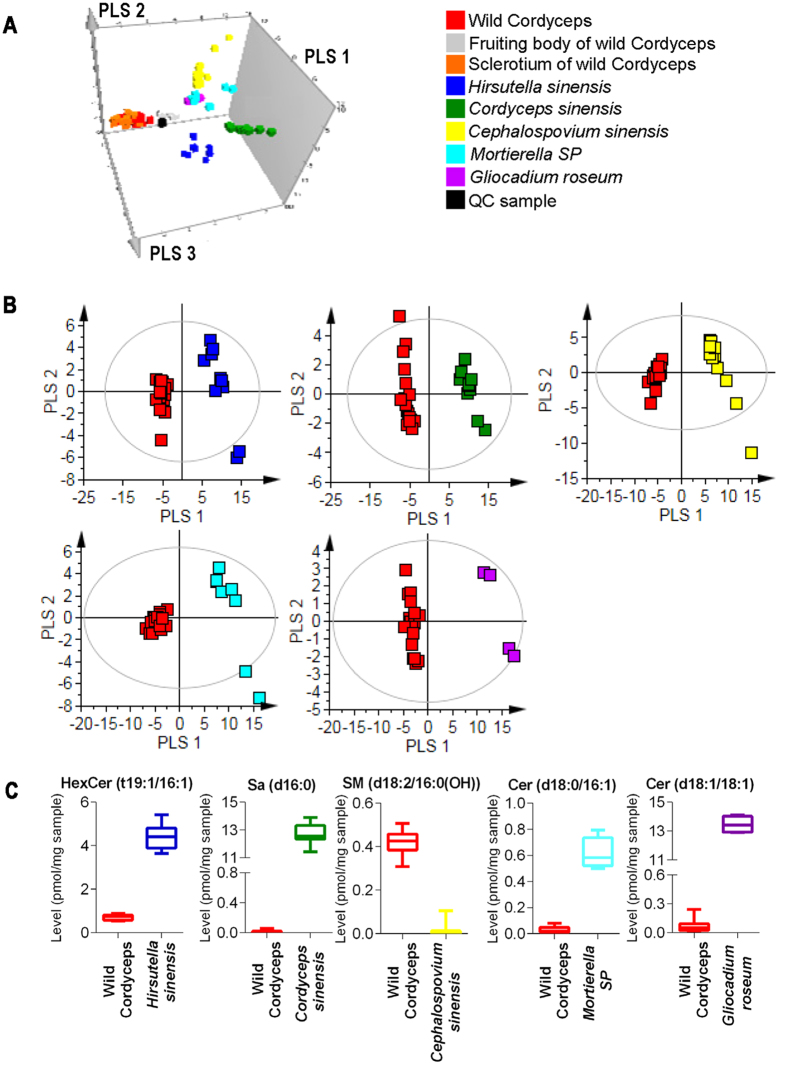
SPL profiles among wild Cordyceps and its mycelia. (**A**) PLS-DA score scatter 3D plot of all samples. (**B**) PLS-DA score scatter plot for wild Cordyceps and its mycelia. (**C**) The significance of representative markers obtained from multivariate statistical analysis among different samples.

The supervised partial least squares discriminant analysis (PLS-DA) model was employed to visualize the general classification of samples. As shown in Fig. 4A, QC samples employed in the analysis were clustered into one group in the PLS-DA score scatter 3D plot. It can be seen that wild Cordyceps was separated from its mycelia at PLS 1 vector, *Hirsutella sinensis* was separated from other mycelia at PLS 2 vector, and *Cordyceps sinensis*, *Cephalospovium sinensis* and other mycelia were separated at PLS 3 vector. PLS-DA was further performed for wild Cordyceps and its mycelia and the plot was shown in Fig. 4B. It can be seen that wild Cordyceps and its mycelia were separated from each other by using this PLS-DA model with a high R^2^Y value of 0.55–0.66 and Q^2^ value of 0.94–0.97. Based on PLS-DA analysis, the SPLs with VIP ≥ 1 were selected as potential markers and further confirmed by using T-test. Finally, potential markers for the classification of wild Cordyceps and *Hirsutella sinensis*, *Cordyceps sinensis*, *Cephalospovium sinensis*, *Mortierella SP*, *Gliocadium roseum* were selected as three SPLs with the highest VIP values, respectively (Table S2). Significant differences of 5 representative markers [HexCer (t19:1/16:1), Sa (d16:0), SM (d18:2/16:0(OH)), Cer (d18:0/16:1) and Cer (d18:1/16:1)] between wild Cordyceps and its mycelia were shown in Fig. 4C. Additionally, potential markers for the classification of different mycelia were found by PLS-DA model analysis (Table S2), in which sphingoid bases was the highest contributing SPL for the classification of most mycelia.

## Discussions

Our study represents the first report for the quantitative profiling of SPLs in wild Cordyceps and its mycelia. By using the fully validated UHPLC-MS method, 101 sphingolipids in wild Cordyceps and its five mycelia were quantitatively profiled. The results revealed a general rank order for the abundance of different classes of sphingolipids in wild Cordyceps and its mycelia. However, remarkable sphingolipid differences between wild Cordyceps and its mycelia were observed. Among five kinds of mycelia, *Cordyceps sinensis* and *Hirsutella sinensis* present quite different profiles as evidenced by particularly high abundance sphingoid bases in *Cordyceps sinensis* and high level of phosphosphingolipids in *Hirsutella sinensis*.

It offers a robust method for evaluating the quality of wild Cordyceps and its mycelia in the aspect of sphingolipidomes. Based on multivariate analysis, respective sphingolipids were found as potential chemical markers for the differentiation of wild Cordyceps and different mycelia. In addition, our study provided comprehensive chemical evidences for the pharmacological effects and clinical efficacy of wild Cordyceps and its mycelia.

## Materials and Methods

### Chemicals

Methanol (LC-MS grade), isopropanol (LC-MS grade) and chloroform (HPLC grade) were purchased from Avantor Performance Materials, Lnc. (Center Valley, PA, USA). Formic acid (LC-MS grade), acetic acid (LC-MS grade), ammonium acetate (purity ≥ 98%) and potassium hydroxide (KOH, purity ≥ 85%) were purchased from Sigma-Aldrich (St. Louis, MO, USA). Distilled water was prepared using a Milli-Q system (Millipore, Billerica, MA). An internal standard cocktail (Internal standards mixture II, 25 μM of each compound in ethanol) containing C17-sphingosine [So (d17:1)], C17-sphinganine [Sa (d17:0)], C17-sphingosine-1-phosphate [S1P (d17:1)], C12-ceramide [Cer (d18:1/12:0)], C12-sphingomyelin [SM (d18:1/12:0)] and C12-glucosylceramide [GlcCer (d18:1/12:0)] was purchased from Avanti Polar Lipids (Alabaster, AL, USA). SPL standards including Cer (d18:1/6:0), Cer (d18:1/18:0), Cer (18:1/22:0), Cer (d18:1/24:0), SM (d18:1/17:0) and SM (d18:1/24:0) were also obtained from Avanti. So (d14:1), So (d20:1) and SM (d18:1/20:0) were obtained from Matreya LLC (Pleasant Gap, PA, USA). Nomenclature of SPL species is conducted according to LIPIDMAPS (Lipidomics Gateway) nomenclature system. For Cer, HexCer, SM, PI-Cer and MIPC, annotation of sphingoid base backbone denotes number of hydroxyl groups, number of total carbon and number of unsaturation degree (e.g. in m18:0, m means one hydroxyl groups; in d18:1, d means two hydroxyl groups; in t18:1, t means three hydroxyl groups). Annotation of N-acyl chain in Cer, HexCer, SM, PI-Cer and MIPC indicates number of total carbon and number of unsaturation degree (e.g. in d18:1/24:0, 24 means number of total carbon; 0 means number of unsaturation degree). For some SMs, annotation of total carbon numbers and total number of unsaturation degree denotes the sum of those of sphingoid base backbone and N-acyl chain.

### Wild Cordyceps and its mycelia

Wild Cordyceps (n = 9) were purchased from Qinghai Province and Wai Yuen Tong Medicine Company Limited (Hong Kong) in August 2013. Each wild Cordyceps included the whole wild Cordyceps and two parts cut from the whole wild Cordyceps [fruiting body of wild Cordyceps (n = 9) and sclerotium of wild Cordyceps (n = 9)]. *Cordyceps sinensis* (n = 5) were from Jiangxi Jiminkexin Pharmaceutical Co., Ltd.; *Hirsutella sinensis* (n = 5) were from Hangzhou Zhongmei Huadong Pharmaceutical Co., Ltd.; *Gliocadium roseum* (n = 2) were from Hebei Changtian Pharmaceutical Co., Ltd.; *Mortierella SP* (n = 4) were from Hangzhou Tianyuan Pharmaceutical Co., Ltd. and Datong Liqun Pharmaceutical Co., Ltd.; *Cephalospovium sinensis* (n = 6) were from Yunnan Baiyao Group Lijiang Pharmaceutical Co. Ltd., Shenyang Dongxin Pharmaceutical Co., Ltd., Hunan Kangerjia Pharmaceutical Co., Ltd., Guizhou Liangji Pharmaceutical Co., Ltd. and Jiangsu Shenhua Pharmaceutical Co., Ltd.

### Preparation of standard and sample solutions

The stock solution of internal standard cocktail (IS 25 μM) was prepared sequentially to afford a series of working solutions (0.05, 0.125, 0.25, 0.5, 1.25, 2.5, 5, 12.5, 25, 37.5 and 50 μM). A mixture standard stock solution was prepared by dissolving the nine standards in methanol to a final concentration of 50 μM. This standard stock solution was then diluted to yield a series of working solutions of 0.5, 1, 2, 5, 10, 25 and 50 μM.

Sample solutions were prepared following the procedures developed in our previous study^20^. Briefly, 35 mg of powdered sample was accurately weighed into a glass bottle. In which extraction solvent 1 [0.75 mL chloroform/methanol (1:2, v/v)] and 10 μL 2.5 μM IS were subsequently added. The mixture was dispersed in ultrasonicator for 30 s and incubated at 48 °C for 12 h. After incubation, 75 μL of KOH in methanol (1 M) was added and the mixture was incubated at 37 °C for 2 h. The resultant extract was then neutralized with acetic acid and centrifuged. The supernatant was collected and the residue was re-extracted with extraction solvent 2 [1 mL chloroform/methanol (2:1, v/v)] and extraction solvent 3 [0.4 mL chloroform/methanol (1:2, v/v), 1 mL chloroform and 2 mL H_2_O], sequentially. The first two supernatants and the third lower layer were combined and N_2_ dried. All extracts were reconstituted in 150 μL methanol. All solutions were filtered through a 0.22 μm filter before analysis.

### LC-MS Conditions

An optimized LC-MS condition established in our lab was employed^20^. Briefly, chromatographic separation was performed on an Agilent 1290 Infinity UHPLC system (Santa Clara, CA, USA) equipped with a binary solvent delivery system and a standard autosampler. An Agilent Eclipse Plus C18 column (100 × 2.1 mm, 1.8 μm) was employed to separate the SPLs. Detection of SPLs was performed on an Agilent 6550 Q-TOF mass spectrometer (Santa Clara, CA, USA). The Jet Stream electrospray ionization source was operated in positive ion mode. Quantitative analysis was carried out in MRM mode using an Agilent 6460 QQQ mass spectrometer (Santa Clara, CA, USA). The MRM transitions (precursor ion **→ **product ion), fragmentor voltages, and CE values selected for each individual SPLs were given in Table 2.

### Method validation

Linearity was determined by spiking 6 IS into samples prior to extraction. Triplicates of each IS were prepared and analyzed at no less than nine appropriate concentration levels. Calibration curves were constructed by linear regression. Linearity was verified by correlation coefficients (r^2^). The lowest concentration in the calibration curve was further diluted for LOQ and LOD tests. LOD and LOQ were defined as the lowest concentration when signal-to-noise ratios (S/N) of about 3 and 10 was obtained, respectively.

The precision of injection was evaluated using 6 replicate injections of the sample solution. The intra- and inter-day precision of the quantitative procedure were determined based on the results of 6 analyses of samples within a day and 9 analyses of samples on three consecutive days. All precision were obtained by calculating the RSDs for the levels of endogenous SPLs in samples.

Recovery was examined by comparing signal response of IS spiked into samples before and after SPLs extraction at three different levels. At each level, six replicates of samples were prepared. Mean recovery and RSDs were calculated to verify the extraction efficiency of IS.

The stability of each analyte in sample solution was tested by measuring the levels directly after sample preparation and at different time intervals, after storage at 4 °C. The stability of system was evaluated using quality control samples (QC, the sample was pooled with equal volume of all analysis samples) which were analyzed across the whole analysis process. All validation parameters were evaluated by calculating RSDs.

The quantitative accuracy of the method was validated by investigating the linear correlation between the added and determined amount of individual SPLs in samples. In the test, a fixed amount of IS (25 pmol) and the amounts of sphingolipid standard varied from 0 to 500 pmol were spiked into a series of samples prior to extraction. The levels of individual SPLs spiked in samples were determined after removal of the pre-determined levels in samples.

### Data processing and statistical analysis

The dynamic MRM data were processed with Agilent Mass Hunter Workstation Software. Quantitative results of SPLs in samples were calculated based on below formula: Level (targeted SPL) = 25 pmol × [Area (targeted SPL)/Area (IS)]. The quantitative data of wild Cordyceps and its mycelia were converted into Microsoft Excel format and imported into SIMCA 14.0 software (version 14.0, Umetrics, Umea, Sweden) for multivariate analysis. Variables significantly changed among different samples were selected based on VIP ≥ 1 and the validated by using T-test analysis.

## Additional Information

**How to cite this article**: Mi, J.-N. *et al.* Quantitative profiling of sphingolipids in wild Cordyceps and its mycelia by using UHPLC-MS. *Sci. Rep.* **6**, 20870; doi: 10.1038/srep20870 (2016).

## Supplementary Material

Supplementary Information

## Figures and Tables

**Table 1:** Calibration equations, correlation coefficients (r^2^), linear ranges, limit of detection (LOD), limit of quantitation (LOQ) and recovery of IS.

IS	Calibration equations	*r*^2^	Linear range (nM)	LOD (nM)	LOQ (nM)	Low^#^ (*n* = 6)		Medium^&^ (*n* = 6)		High^*^ (*n* = 6)		Mean Recovery (%)
						Recovery (%)	RSD (%)	Recovery (%)	RSD (%)	Recovery (%)	RSD (%)	
IS-1	Y = 443.61X – 1920.10	0.9992	3.34–3340.00	0.025	0.835	96.72	3.50	83.13	1.83	81.31	2.37	87.05
IS-2	Y = 86.64X – 960.46	0.9977	8.35–3340.00	1.670	6.680	90.23	3.56	89.33	1.28	71.59	1.61	83.72
IS-3	Y = 92.01X – 1303.30	0.9977	16.70–3340.00	3.340	16.700	88.98	4.81	75.43	2.30	80.69	1.27	81.70
IS-4	Y = 413.61X – 12809.00	0.9988	8.35–3340.00	0.025	1.670	104.12	3.07	101.40	4.46	94.94	1.39	100.15
IS-5	Y = 1171.30X + 12114.00	0.9994	3.34–3340.00	0.005	0.017	100.65	2.99	97.01	2.10	100.60	1.62	99.42
IS-6	Y = 74.38X – 2785.00	0.9983	8.35–3340.00	1.670	6.680	73.06	3.24	94.43	2.28	97.81	4.84	88.43

^#^Low, Low concentration; ^&^Medium, Medium concentration; *High, High concentration.

**Table 2:** MRM parameters and validation data for the quantitation of 101 SPLs in wild Cordyceps and its mycelia.

Class	Subclass	No.	Name	RT (min)	MRM transitions (*m/z*)	Fragmentor (v)	CE^*^ (v)	Precision and stability (RSD%)^£^				
								Injection precision	Intra-day precision	Inter-day precision	Analyte’s stability	System’s stability
Sphingoid bases	So	**IS-1**	**So (d17:1)**	**6.37**	**286.3** → **268.3**	**80**	**5**	**1.17**	**1.25**	**2.02**	**2.04**	**2.88**
		1	So (d14:2)	3.76	242.2 → 224.2	80	5	ND^**$**^	ND	ND	ND	2.51
		2	So (d14:1)	5.30	244.2 → 226.1	80	5	0.74	2.85	2.18	2.50	2.43
		3	So (d16:1)	5.95	272.3 → 254.2	80	5	ND	ND	ND	ND	4.68
		4	So (d17:2)	7.11	284.3 → 266.2	80	5	ND	ND	ND	ND	3.69
		5	So (d18:2)	6.50	298.3 → 280.3	80	5	0.98	5.08	6.59	3.97	2.45
		6	So (d18:1)	6.78	300.3 → 282.3	80	5	0.41	1.85	2.10	1.90	2.99
		7	So (t18:1)	6.38	316.3 → 298.3	80	5	0.55	1.16	1.09	2.69	2.30
		8	So (t18:1) isomer	6.90	316.3 → 298.3	80	5	0.52	1.19	1.43	4.40	2.35
		9	So (d20:1)	9.41	328.3 → 310.3	80	5	0.35	1.80	1.86	2.09	6.36
		10	So (t22:2)	9.13	370.3 → 352.3	80	5	1.15	12.19	14.89	1.57	3.37
	S1P	**IS-2**	**S1P (d17:1)**	**6.55**	**366.2** → **250.3**	**105**	**10**	**0.30**	**0.94**	**1.74**	**4.79**	**5.40**
		11	S1P (d18:1)	9.03	380.3 → 362.3	105	10	1.02	4.67	2.43	2.54	5.76
	S1Po^#^	12	S1Po (d18:1)	6.55	467.4 → 449.3	105	10	ND	ND	ND	ND	5.83
	Sa	**IS-3**	**Sa (d17:0)**	**6.57**	**288.3** → **270.3**	**110**	**20**	**0.49**	**2.15**	**2.19**	**1.37**	**4.34**
		13	Sa (d16:0)	6.24	274.3 → 256.3	110	20	ND	ND	ND	ND	4.51
		14	Sa (m18:0)	7.12	286.3 → 268.3	110	20	ND	ND	ND	ND	3.72
		15	Sa (d17:0) isomer	5.51	288.3 → 270.3	110	20	ND	ND	ND	ND	3.31
		16	Sa (d18:0)	6.91	302.3 → 284.3	110	20	0.63	2.58	3.41	4.02	3.88
		17	Sa (t18:0)	6.61	318.3 → 300.3	110	20	1.03	0.57	0.61	0.83	4.48
		18	Sa (t18:0) isomer	6.32	318.3 → 300.3	110	20	0.58	2.43	2.06	6.09	3.11
Ceramides	Cer	**IS-4**	**Cer (d18:1/12:0)**	**10.96**	**482.5** → **264.3**	**130**	**25**	**1.88**	**3.92**	**4.03**	**3.57**	**5.41**
		19	Cer (d14:1/22:0)	14.64	566.5 → 208.2	130	25	1.32	3.48	3.47	11.06	12.46
		20	Cer (d18:1/2:0)	9.04	342.3 → 264.3	130	25	5.60	12.69	11.98	4.50	4.86
		21	Cer (d18:1/14:2)	11.06	506.5 → 264.3	130	25	0.53	3.79	3.89	4.72	10.21
		22	Cer (d18:1/14:1)	11.15	508.5 → 264.3	130	25	2.99	7.11	9.99	16.17	5.95
		23	Cer (d18:1/14:0)	11.85	510.5 → 264.3	130	25	2.17	4.63	4.19	18.05	10.86
		24	Cer (d18:1/15:0)	12.33	524.5 → 264.3	130	25	0.50	1.55	1.36	9.58	14.48
		25	Cer (d18:1/16:2)	11.84	534.5 → 264.3	130	25	2.43	3.31	3.83	12.62	10.23
		26	Cer (d18:1/16:1)	12.43	536.5 → 264.3	130	25	0.54	1.26	3.14	4.72	9.47
		27	Cer (d18:1/16:0)	12.92	538.5 → 264.3	130	25	1.50	2.80	6.05	9.14	11.30
		28	Cer (d18:1/17:1)	13.03	550.5 → 264.3	130	25	1.78	4.85	4.33	15.91	13.69
		29	Cer (d18:1/18:2)	13.45	562.5 → 264.3	130	25	1.12	5.98	6.29	7.52	14.02
		30	Cer (d18:1/18:1)	13.57	564.5 → 264.3	130	25	0.78	0.95	1.20	4.06	9.26
		31	Cer (d18:1/18:0)	14.36	566.5 → 264.3	130	25	1.72	3.04	4.54	8.21	12.44
		32	Cer (d18:1/20:1)	15.86	592.6 → 264.3	130	25	1.01	8.45	7.15	6.96	20.58
		33	Cer (d18:1/22:0)	18.20	622.6 → 264.3	130	25	4.88	10.12	8.26	17.27	19.05
		34	Cer (d18:1/23:0)	19.10	636.6 → 264.3	130	25	2.82	4.01	7.49	5.38	14.47
		35	Cer (d18:1/24:0)	20.10	650.6 → 264.3	130	25	4.22	4.78	4.05	3.73	13.24
		36	Cer (d18:1/25:0)	19.25	664.7 → 264.3	130	25	ND	ND	ND	ND	14.40
		37	Cer (d18:1/26:1)	19.23	676.7 → 264.3	130	25	5.08	2.38	2.94	7.04	14.17
		38	Cer (d18:2/15:0)	11.83	522.5 → 262.3	130	25	2.53	4.35	6.87	14.73	11.54
		39	Cer (d18:2/16:1)	11.85	534.5 → 262.3	130	25	0.73	6.26	3.63	7.28	2.68
		40	Cer (d18:2/16:0)	12.38	536.5 → 262.3	130	25	0.39	1.49	1.17	3.74	11.23
		41	Cer (d18:2/18:1)	13.50	562.5 → 262.3	130	25	ND	ND	ND	ND	10.07
		42	Cer (d18:2/23:0)	18.18	634.6 → 262.3	130	25	ND	ND	ND	ND	21.35
		43	Cer (d18:2/24:0)	19.06	648.6 → 262.3	130	25	4.01	4.34	5.62	5.10	13.95
		44	Cer (d19:2/16:0)	12.76	550.5 → 276.3	130	25	1.04	1.50	2.49	5.83	10.33
		45	Cer (d18:1/16:1(OH))	12.32	552.5 → 264.3	130	25	3.06	4.12	4.46	9.65	14.78
		46	Cer (d18:1/16:0(OH))	12.40	554.5 → 264.3	130	25	1.34	0.73	0.71	6.06	11.96
		47	Cer (d18:1/17:1(OH))	15.36	566.5 → 264.3	130	25	2.36	2.68	3.95	6.35	14.16
		48	Cer (d18:1/18:1(OH))	14.53	580.5 → 264.3	130	25	0.31	2.22	3.70	4.98	14.72
		49	Cer (d18:1/18:0(OH))	13.55	582.5 → 264.3	130	25	ND	ND	ND	ND	8.01
		50	Cer (d18:1/19:1(OH))	12.55	594.5 → 264.3	130	25	1.66	2.36	2.18	7.49	12.06
		51	Cer (d18:1/22:0(OH))	17.11	638.6 → 264.3	130	25	2.40	6.06	10.11	19.27	19.71
		52	Cer (d18:2/16:0(OH))	11.84	552.5 → 262.3	130	25	2.88	5.74	14.77	19.71	13.98
		53	Cer (d18:2/18:1(OH))	11.52	578.5 → 262.3	130	25	ND	ND	ND	ND	12.92
		54	Cer (d19:2/16:0(OH))	12.20	566.5 → 276.3	130	25	0.35	3.57	5.49	2.41	6.30
		55	Cer (d18:0/15:0)	12.75	526.5 → 266.3	130	25	1.03	7.52	7.17	15.16	12.59
		56	Cer (d18:0/16:1)	13.23	538.5 → 266.3	130	25	2.35	5.60	4.32	10.49	9.24
		57	Cer (d18:0/16:0)	13.35	540.5 → 266.3	130	25	1.77	5.60	4.49	11.40	9.75
		58	Cer (d18:0/18:0)	14.63	568.6 → 266.3	130	25	ND	ND	ND	ND	14.79
		59	Cer (d18:0/26:0)	20.82	680.7 → 266.3	130	25	ND	ND	ND	ND	14.20
		60	Cer (t18:1/18:0)	12.99	582.5 → 262.3	130	25	ND	ND	ND	ND	14.17
		61	Cer (t18:1/22:1)	16.15	636.6 → 262.3	130	25	ND	ND	ND	ND	14.48
		62	Cer (t18:1/22:0)	15.64	638.6 → 262.3	130	25	5.95	22.80	21.75	17.75	18.08
		63	Cer (t18:0/16:0)	12.28	556.5 → 264.3	130	25	1.02	3.71	4.67	9.28	10.46
		64	Cer (t18:0/22:1)	16.12	638.6 → 264.3	130	25	ND	ND	ND	ND	21.05
		65	Cer (t18:0/22:0)	16.13	640.6 → 264.3	130	25	0.43	9.40	8.70	7.21	14.36
		66	Cer (t18:0/24:0)	17.45	668.7 → 264.3	130	25	1.76	2.15	2.66	4.83	21.36
		67	Cer (t18:0/18:0(OH))	12.98	600.6 → 264.3	130	25	2.68	4.36	4.53	11.48	12.06
		68	Cer (t18:0/22:0(OH))	15.48	656.6 → 264.3	130	25	1.17	3.70	2.89	5.04	14.51
		69	Cer (t18:0/24:0(OH))	16.83	684.7 → 264.3	130	25	2.32	2.59	2.43	18.20	22.32
Glycosphingolipids	HexCer	**IS-5**	**GlcCer (d18:1/12:0)**	**10.40**	**644.5** → **264.3**	**130**	**30**	**1.99**	**1.09**	**1.04**	**4.79**	**5.89**
		70	HexCer (d18:1/16:0)	12.21	700.6 → 264.3	130	30	1.53	6.73	9.43	7.96	14.79
		71	HexCer (d18:2/16:0)	11.52	698.6 → 262.3	130	30	1.20	2.91	2.01	4.81	4.81
		72	HexCer (d18:2/16:0(OH))	11.25	714.6 → 262.3	130	30	2.15	6.51	7.11	5.81	14.71
		73	HexCer (d19:2/15:0)	11.46	698.6 → 276.3	130	30	1.93	6.63	5.60	5.33	6.50
		74	HexCer (d19:2/16:0(OH))	11.75	728.6 → 276.3	130	30	0.50	11.97	15.09	4.32	8.65
		75	HexCer (d19:2/17:0(OH))	12.00	742.6 → 276.3	130	30	5.44	2.33	2.42	23.88	20.74
		76	HexCer (d19:2/18:0(OH))	12.53	756.6 → 276.3	130	30	0.58	3.80	3.08	12.18	10.77
		77	HexCer (t19:1/16:1)	11.20	728.6 → 276.3	130	30	ND	ND	ND	ND	10.09
Phosphosphingolipids	SM	**IS-6**	**SM (d18:1/12:0)**	**10.37**	**647.5** → **184.1**	**170**	**20**	**1.87**	**2.02**	**1.72**	**5.38**	**5.40**
		78	SM (d18:1/14:0)	11.1	675.5 → 184.1	170	20	0.39	2.83	3.51	2.44	3.63
		79	SM (d18:1/16:0)	12.00	703.6 → 184.1	170	20	0.52	1.33	2.18	2.3	4.9
		80	SM (d18:1/18:0)	13.25	731.6 → 184.1	170	20	0.83	1.94	2.06	3.6	4.99
		81	SM (d18:1/20:0)	14.51	759.6 → 184.1	170	20	1.06	5.48	4.76	5.08	10.22
		82	SM (d18:1/22:0)	15.61	787.7 → 184.1	170	20	5.52	4.99	4.81	2.1	11.33
		83	SM (d18:1/24:0)	16.91	815.7 → 184.1	170	20	2.12	3.65	2.42	2.09	14.39
		84	SM (d18:1/16:1)	11.66	701.6 → 184.1	170	20	1.07	2.15	2.05	3.98	3.62
		85	SM (d18:1/20:1)	13.90	757.6 → 184.1	170	20	1.75	2.8	3.48	3.14	9.43
		86	SM (d18:1/22:1)	15.12	785.7 → 184.1	170	20	0.47	4.6	6.32	4.76	7.39
		87	SM (d18:2/18:0)	12.75	729.6 → 184.1	170	20	1.4	6.59	6.9	7.41	5.37
		88	SM (d18:2/18:1)	11.81	727.6 → 184.1	170	20	ND	ND	ND	ND	7.42
		89	SM (d18:1/18:1(OH))	12.38	745.6 → 184.1	170	20	1.35	2.82	3.91	5.21	7.03
		90	SM (d18:2/16:0(OH))	11.07	717.6 → 184.1	170	20	1.43	1.33	0.86	4.21	4.13
		91	SM (d33:1)	11.56	689.6 → 184.1	170	20	2.79	5.38	4.6	2.57	7.44
		92	SM (d39:2)	14.56	771.6 → 184.1	170	20	2.68	5.07	4.16	4.51	11.93
		93	SM (d42:2)	16.14	813.7 → 184.1	170	20	1.65	2.82	2.77	1.49	10.5
		94	SM (d34:0)	12.5	705.6 → 184.1	170	20	1.29	5.33	3.96	6.85	6.14
		95	SM (d36:0)	13.84	733.6 → 184.1	170	20	1.91	3.23	3.04	5.74	7.85
		96	SM (d38:0)	14.90	761.7 → 184.1	170	20	ND	ND	ND	ND	11.75
	PI-Cer^#^	97	PI-Cer (d18:0/16:0(OH))	10.90	798.5 → 538.5	170	30	ND	ND	ND	ND	5.50
		98	PI-Cer (t18:0/22:0(OH))	12.84	898.6 → 638.6	170	30	ND	ND	ND	ND	14.83
		99	PI-Cer (t18:0/24:0(OH))	13.95	926.7 → 666.6	170	30	1.73	5.26	4.66	12.65	13.32
	MIPC^#^	100	MIPC (t18:0/22:0(OH))	12.60	1060.7 → 638.6	170	40	ND	ND	ND	ND	20.32
		101	MIPC (t18:0/24:0(OH))	13.68	1088.7 → 666.6	170	40	2.37	19.15	23.55	20.13	11.82

^*^CE, Collision Energy. ^£^Precisions and stabilities of endogenous SPLs in validation samples (*Hirsutella sinensis*) were calculated according to the quantitative data, while precisions and stabilities of 6 IS were calculated using peak area data; system’s stability was determined using QC sample. ^$^ ND, Not detected in validation samples. ^#^ The IS for quantitation of S1Po was IS-2; the IS for quantitation of PI-Cer and MIPC was IS-4.
