# Supplementary material for: Quantitative profiling of sphingolipids in wild Cordyceps and its mycelia by using UHPLC-MS
Source: Sci Rep. 2016 Feb 12;6:20870. doi: 10.1038/srep20870 (PMC4751452; doi:10.1038/srep20870)

Supplementary Information

Quantitative profiling of sphingolipids in wild Cordyceps and its mycelia by using UHPLC-MS

Jia-Ning Mi, Jing-Rong Wang, Zhi-Hong Jiang

Table S1. Identification of SPLs in wild Cordyceps and its mycelia by using UHPLC-QTOF-MS/MS

No.	Name	Formula	$[M+H]^+$ (*m/z*)	Diff (ppm)	Score	RT (min)	ms/ms fragments (*m/z*)
**IS-1**	**So (d17:1)**	**C17H35NO2**	**286.2740**	**0.06**	**99.70**	**6.37**	**268.2627, 250.2545**
1	So (d14:2)	C14H27NO2	242.2111	-0.82	83.88	3.76	224.1996, 206.1895, 194.1887, 60.0440
2	So (d14:1)	C14H29NO2	244.2270	-1.34	81.57	5.30	226.1296, 60.0436
3	So (d16:1)	C16H33NO2	272.2578	-2.50	91.98	5.95	254.2414, 236.2334, 60.0443
4	So (d17:2)	C17H33NO2	284.2583	1.30	49.53	7.11	266.2485
5	So (d18:2)	C18H35NO2	298.2742	-3.82	83.03	9.10	280.2652, 60.0448
6	So (d18:1)	C18H37NO2	300.2898	-0.05	99.57	8.07	282.2774, 264.2371, 60.0450
7	So (t18:1)	C18H37NO3	316.2850	-1.38	82.52	6.38	298.2727, 280.2623, 262.2517, 250.2525, 60.0446
8	So (t18:1) isomer	C18H37NO3	316.2847	-0.28	81.81	6.90	298.2746, 60.0444
9	So (d20:1)	C20H41NO2	328.3213	0.64	98.97	9.41	310.3100
10	So (t22:2)	C22H43NO3	370.3316	-0.35	98.90	7.49	352.3225
**IS-2**	**S1P (d17:1)**	**C17H36NO5P**	**366.2406**	**-0.56**	**99.74**	**6.55**	**250.2536**
11	S1P (d18:1)	C18H38NO5P	380.2564	1.20	82.71	9.03	362.3209, 264.2659
12	S1Po (d18:1)*	C23H49N2O5P	467.3597	-5.08	74.93	6.55	449.3489, 184.0734
**IS-3**	**Sa (d17:0)**	**C17H37NO2**	**288.2898**	**-0.09**	**99.29**	**6.57**	**270.2781, 252.2673, 240.2680, 60.0442**
13	Sa (d16:0)	C16H35NO2	274.2740	-0.68	95.38	6.24	256.2617, 238.2522, 60.0443
14	Sa (m18:0)	C18H39NO	286.3105	0.19	98.89	7.12	268.2989
15	Sa (d17:0) isomer*	C17H37NO2	288.2889	-3.54	95.85	5.51	270.2784
16	Sa (d18:0)	C18H39NO2	302.3048	-1.81	46.59	6.91	284.2926, 266.2819, 254.2842, 60.0439
17	Sa (t18:0)	C18H39NO3	318.3026	7.56	84.09	6.61	300.2883, 282.2782, 264.2683, 270.2782, 252.2670, 60.0442
18	Sa (t18:0) isomer	C18H39NO3	318.3004	-0.40	99.04	6.32	300.2888, 288.2772, 270.2790
**IS-4**	**Cer (d18:1/12:0)**	**C30H59NO3**	**482.4565**	**-1.59**	**92.99**	**10.96**	**464.4431, 446.4281, 434.4308, 282.2786, 264.2678, 252.2671**
19	Cer (d14:1/22:0)	C36H71NO3	566.5468	-6.53	55.89	14.64	548.5263, 530.5278, 518.5263, 226.2156,

							208.2054, 196.2049, 60.0442
20	Cer (d18:1/2:0)*	C20H39NO3	342.3000	1.50	91.72	9.04	264.2606
21	Cer (d18:1/14:2)	C32H59NO3	506.4574	0.06	93.77	11.06	282.2837, 264.2675, 60.0617
22	Cer (d18:1/14:1)	C32H61NO3	508.4715	-1.59	96.56	11.15	264.2723
23	Cer (d18:1/14:0)	C32H63NO3	510.4878	-1.20	96.55	11.85	492.4738, 474.4603, 462.4662, 282.2777, 264.2673, 252.2662, 60.0435
24	Cer (d18:1/15:0)	C33H65NO3	524.5042	0.39	97.66	12.33	506.4927, 488.4815, 282.2796, 264.2687, 252.2678, 60.0444
25	Cer (d18:1/16:2)	C34H63NO3	534.4880	0.38	97.77	11.84	282.2789, 264.2685, 252.2686
26	Cer (d18:1/16:1)	C34H65NO3	536.5042	0.36	94.85	12.43	518.493, 506.4931, 488.4800, 282.2795, 264.2691, 252.2691, 60.0447
27	Cer (d18:1/16:0)	C34H67NO3	538.5204	1.72	98.10	12.92	520.4758, 502.4667, 282.2624, 264.2530, 252.2530, 60.0412
28	Cer (d18:1/17:1)	C35H67NO3	550.5185	-0.65	98.47	13.03	300.2898, 282.2742, 264.2661, 60.0437
29	Cer (d18:1/18:2)	C36H67NO3	562.5183	-1.90	97.30	13.45	544.5042, 300.2883, 282.2783, 264.2674, 252.2687, 60.0446
30	Cer (d18:1/18:1)	C36H69NO3	564.5353	0.50	99.81	13.57	548.5343, 530.4838, 300.2836, 282.2760, 264.2643, 252.2700, 60.0442
31	Cer (d18:1/18:0)	C36H71NO3	566.5486	-2.80	67.90	14.36	548.5343, 518.5343, 282.2788, 264.2708, 252.2720, 60.0450
32	Cer (d18:1/20:1)	C38H73NO4	592.5713	7.76	73.80	15.86	556.5546, 282.2793, 264.2689, 252.2685, 60.0443
33	Cer (d18:1/22:0)	C40H79NO3	622.6127	-1.42	96.67	18.20	604.5998, 586.5841, 300.2889, 282.2779, 264.2680, 252.2672, 60.0441
34	Cer (d18:1/23:0)*	C41H81NO3	636.6274	-2.36	94.37	19.10	618.6192, 600.6006, 300.2886, 282.2778, 264.2695, 252.2668, 60.0441
35	Cer (d18:1/24:0)*	C42H83NO3	650.6433	-2.38	94.72	20.10	632.6333, 614.6204, 300.2887, 282.2806, 264.2690, 252.2727, 60.0441
36	Cer (d18:1/25:0)	C43H85NO3	664.6585	-3.59	74.25	19.25	300.2901, 282.2772, 264.2687, 252.2648,

							60.0434
37	Cer (d18:1/26:1)	C44H85NO3	676.6589	-2.30	72.91	19.23	300.2924, 282.2786, 264.2682, 252.2685, 60.0435
38	Cer (d18:2/15:0)	C33H63NO3	522.4881	-0.99	96.90	11.83	504.4757, 486.4644, 474.4622, 280.2621, 262.2525, 250.2525, 60.0437
39	Cer (d18:2/16:1)	C34H63NO3	534.4887	1.13	46.93	11.85	516.4747, 280.2627, 262.2520, 250.2527, 60.0448
40	Cer (d18:2/16:0)	C34H65NO3	536.5047	1.63	98.33	12.38	518.5032, 500.4807, 488.4866, 280.2630, 262.2528, 250.2523, 60.0446
41	Cer (d18:2/18:1)	C36H67NO3	562.5183	-1.90	97.30	13.50	544.5027, 298.2732, 280.2608, 262.2517, 60.0443
42	Cer (d18:2/23:0)	C41H79NO3	634.6120	-2.61	90.40	18.18	616.6017, 598.6093, 298.2733, 280.2624, 262.2523, 250.2508, 60.0446
43	Cer (d18:2/24:0)*	C42H81NO3	648.6282	-1.20	96.21	19.06	630.6151, 612.5993, 298.2729, 280.2626, 262.2522, 250.2522, 60.0443
44	Cer (d19:2/16:0)	C35H67NO3	550.5180	-2.66	95.77	12.76	532.5053, 514.4940, 502.4963, 294.2781, 276.2676, 264.2679, 60.0441
45	Cer (d18:1/16:1(OH))	C34H65NO4	552.4987	0.18	93.34	12.32	534.4662,516.4655, 282.2793, 264.2690, 252.2664, 60.0434
46	Cer (d18:1/16:0(OH))	C34H67NO4	554.5137	-1.09	98.26	12.40	536.4763, 518.4844, 500.4834, 318.2981, 300.2877, 282.2777, 264.2676, 252.2678, 60.0442
47	Cer (d18:1/17:1(OH))	C35H67NO4	566.5138	-0.88	97.50	15.36	548.5389, 530.5290, 300.2885, 282.2786, 264.2678, 252.2676, 60.0442
48	Cer (d18:1/18:1(OH))	C36H69NO4	580.5215	1.79	89.58	14.53	562.5497, 532.3500, 282.2766, 264.2688, 252.2640, 60.0421
49	Cer (d18:1/18:0(OH))	C36H71NO4	582.5455	0.20	85.70	13.55	564.2850, 534.1585, 282.2805, 264.2682, 252.2677, 60.0442
50	Cer (d18:1/19:1(OH))	C37H71NO4	594.5424	-4.67	81.99	12.55	282.2656, 264.2670, 252.2348,60.0452

51	Cer (d18:1/22:0(OH))*	C40H79NO4	638.6089	1.10	98.65	17.11	620.5936, 602.5772, 300.2884, 282.2769, 264.2676, 252.2644, 60.0443
52	Cer (d18:2/16:0(OH))	C34H65NO4	552.4991	0.91	84.54	11.84	534.4955, 516.4797, 298.2779, 280.2621, 262.2527, 250.2495, 60.0449
53	Cer (d18:2/18:1(OH))	C36H67NO4	578.5137	-1.04	88.31	11.52	560.5017, 542.4887, 524.4798, 512.4791, 316.2824, 298.2728, 280.2623, 262.2518, 250.2508, 60.0441
54	Cer (d19:2/16:0(OH))	C35H67NO4	566.5167	3.22	90.99	12.20	566.5060, 548.5021, 530.4916, 294.2780, 276.2684, 264.2693, 60.0436
55	Cer (d18:0/15:0)*	C33H67NO3	526.5185	-1.58	49.26	12.75	508.5046, 490.4941, 284.2962, 266.2840, 254.2827, 60.0441
56	Cer (d18:0/16:1)	C34H67NO3	538.5194	0.01	94.87	13.23	520.4573, 502.4465, 302.2804, 284.2702, 266.2606, 254.2210, 60.0398
57	Cer (d18:0/16:0)	C34H69NO3	540.5350	0.02	47.62	13.35	522.5168, 504.5167, 302.3034, 284.2930, 266.2819, 254.2848, 60.0442
58	Cer (d18:0/18:0)	C36H73NO3	568.5660	-0.53	97.48	14.63	550.5549, 532.5356, 302.3041, 284.2927, 266.2838, 254.2854, 60.0439
59	Cer (d18:0/26:0)*	C44H89NO3	680.6932	2.63	95.31	20.82	662.6779, 644.6688, 302.3044, 284.2943, 266.2837, 254.2835, 60.0443
60	Cer (t18:1/18:0)	C36H71NO4	582.5454	-2.78	87.05	12.99	564.5238, 546.5252, 280.2609, 262.2535, 250.2525, 60.044
61	Cer (t18:1/22:1)*	C40H77NO4	636.5916	-1.41	97.00	16.15	618.5775, 600.5669, 316.2958, 298.2726, 280.2620, 262.2527, 250.2512, 60.0442
62	Cer (t18:1/22:0)	C40H79NO4	638.6075	-1.72	95.50	15.64	620.5942, 602.5830, 584.5657, 316.2827, 298.2730, 280.2626, 262.2522, 250.2507, 60.0443
63	Cer (t18:0/16:0)	C34H69NO4	556.5283	-3.49	47.41	12.28	282.2782, 264.2673, 252.2681, 60.0443
64	Cer (t18:0/22:1)	C40H79NO4	638.6066	-2.51	92.94	16.12	620.5912, 602.5785, 318.2885, 282.2785, 264.2677, 252.2681, 60.0443

65	Cer (t18:0/22:0)	C40H81NO4	640.6224	-2.61	95.18	16.13	622.6117, 604.6000, 318.3000, 300.2895, 282.2789, 264.2680, 252.2677, 60.0445
66	Cer (t18:0/24:0)	C42H85NO4	668.6558	1.30	98.39	17.45	650.6451, 632.6331, 614.6217, 318.3005, 300.2900, 282.2792, 264.2687, 252.2685, 60.0446
67	Cer (t18:0/18:0(OH))	C36H73NO5	600.5558	-1.30	96.81	12.98	582.5451, 564.5357, 546.5223, 534.5204, 318.3015, 300.2895, 282.2799, 264.2693, 252.2686, 60.045
68	Cer (t18:0/22:0(OH))	C40H81NO5	656.6183	-1.13	97.91	15.48	638.6032, 620.5948, 602.5856, 318.2980, 300.2891, 282.2780, 264.2676, 252.2673, 60.0441
69	Cer (t18:0/24:0(OH))	C42H85NO5	684.6709	3.48	52.12	16.83	666.6481, 648.6362, 630.6249, 612.6156, 318.3042, 300.2934, 282.2830, 264.2720, 252.2713, 60.0454
**IS-5**	**GlcCer (d18:1/12:0)**	**C36H69NO8**	**644.5116**	**-0.11**	**99.98**	**10.40**	**264.2684, 60.0441**
70	HexCer (d18:1/16:0)*	C40H77NO8	700.5704	-2.69	93.97	12.21	682.5608, 664.5436, 520.5076,502.4982, 490.4893,282.2788, 264.2683, 252.2698,60.0445
71	HexCer (d18:2/16:0)	C40H75NO8	698.5573	1.27	86.87	11.52	680.5461, 536.5047, 518.4944, 500.4838, 280.2669, 262.2535, 250.2535
72	HexCer (d18:2/16:0(OH))	C40H75NO9	714.5522	0.55	97.12	11.25	696.5392, 534.4879, 516.4774, 504.477, 280.2636, 262.2532, 250.2534, 60.0455
73	HexCer (d19:2/15:0)	C40H75NO8	698.5555	-1.43	94.75	11.46	680.4951, 662.6058, 536.4120, 518.4675, 294.2805, 276.2660, 264.2522, 60.0440
74	HexCer (d19:2/16:0(OH))	C41H77NO9	728.5679	1.05	86.31	11.75	710.5562, 692.5422, 566.5138, 548.5038, 530.4929, 518.4936, 294.2794, 276.2691, 264.2694, 60.0447
75	HexCer (d19:2/17:0(OH))*	C42H79NO9	742.5842	1.87	94.85	12.00	724.5716, 562.5191, 544.5080, 532.5108, 294.2787, 276.2696, 60.0451

76	HexCer (d19:2/18:0(OH))	C43H81NO9	756.5992	0.21	96.54	12.53	738.5863, 576.5335, 558.5236, 546.5249, 294.2788, 276.2689, 264.2686, 60.0447
77	HexCer (t19:1/16:1)	C41H77NO9	728.5678	0.82	98.38	11.20	710.5435, 692.5330, 548.4912, 530.4811, 512.4701, 330.2718, 312.2484, 294.2775, 276.2677, 60.0441
**IS-6**	**SM (d18:1/12:0)**	**C35H71N2O6P**	**647.5119**	**2.88**	**89.65**	**10.37**	**282.2788, 264.2672, 184.0742**
78	SM (d18:1/14:0)	C37H75N2O6P	675.5435	-0.31	99.53	11.10	264.2628, 184.0733
79	SM (d18:1/16:0)	C39H79N2O6P	703.5773	3.43	87.95	12.00	264.2723, 184.0738
80	SM (d18:1/18:0)	C41H83N2O6P	731.6061	0.01	99.51	13.25	264.2670, 184.0732
81	SM (d18:1/20:0)	C43H87N2O6P	759.6377	0.06	99.42	14.51	264.2675, 184.0731
82	SM (d18:1/22:0)	C45H91N2O6P	787.6679	-1.82	93.87	15.61	264.2642, 184.074
83	SM (d18:1/24:0)	C47H95N2O6P	815.6937	-7.51	66.52	16.91	264.2616, 184.0717
84	SM (d18:1/16:1)	C39H77N2O6P	701.5586	-0.56	99.03	11.66	264.2670, 184.0733
85	SM (d18:1/20:1)	C43H85N2O6P	757.6234	2.11	95.74	13.90	264.2676, 184.0726
86	SM (d18:1/22:1)	C45H89N2O6P	785.6519	-2.62	85.23	15.12	264.2686, 184.0741
87	SM (d18:2/18:0)	C41H81N2O6P	729.5895	-2.38	81.30	12.75	262.2550, 184.0738
88	SM (d18:2/18:1)	C41H79N2O6P	727.5733	-3.23	89.12	11.81	262.2520, 184.0729
89	SM (d18:1/18:1(OH))	C41H81N2O7P	745.5853	-0.49	94.97	12.38	264.2709, 184.0737
90	SM (d18:2/16:0(OH))	C39H77N2O7P	717.5546	0.34	70.39	11.07	262.2531, 184.0738
91	SM (d33:1)	C38H77N2O6P	689.5578	-1.61	93.11	11.56	184.0728
92	SM (d39:2)	C44H87N2O6P	771.6349	-4.65	72.19	14.56	184.0726
93	SM (d42:2)	C47H93N2O6P	813.6809	-5.03	78.04	16.14	184.0734
94	SM (d34:0)	C39H81N2O6P	705.5906	0.19	99.72	12.50	184.0732
95	SM (d36:0)	C41H85N2O6P	733.6209	-1.32	96.37	13.84	184.0734
96	SM (d38:0)	C43H89N2O6P	761.6523	-1.98	91.96	14.90	184.0728
97	PI-Cer (d18:0/16:0(OH))*	C40H80NO12P	798.5494	0.38	91.30	10.90	780.5319,556.5266, 538.5169, 520.5078, 284.2930, 266.2852,60.0425
98	PI-Cer (t18:0/22:0(OH))	C46H92NO13P	898.6364	-1.67	96.77	12.84	880.6528, 666.6358, 638.6056, 620.5907, 602.4465, 300.2888, 282.2773, 264.2655,

							252.2662,60.0443
99	PI-Cer (t18:0/24:0(OH))	C48H96NO13P	926.6692	0.01	98.13	13.95	908.6490, 684.6483, 666.6362, 648.6261, 630.6105, 300.2882, 282.2777, 264.2674, 252.2677, 60.0442
100	MIPC (t18:0/22:0(OH))	C52H102NO18P	1060.6880	-2.55	91.03	12.60	1042.5295, 898.6364, 880.6253, 656.6104, 638.6042, 300.2909, 282.2738, 264.2668, 60.0447
101	MIPC (t18:0/24:0(OH))	C54H106NO18P	1088.7205	-1.36	95.15	13.68	926.9604, 666.6345, 648.6163, 300.2877, 282.2777, 264.2664, 60.0445

* Sphingolipids were identified from mycelia only.

Table S2. The level of representative markers discriminating wild Cordyceps and its mycelia.

Group A V.S. Group B	SPLs	VIP	Group A	Group B	*p*
Wild Cordyceps V.S. *Hirsutella sinensis*	HexCer (t19:1/16:1)	1.38	0.715 ±0.120	4.416 ±0.619	0.0000
	HexCer (d19:2/16:0(OH))	1.37	0.958 ±0.289	5.436 ±0.638	0.0000
	So (d18:1)	1.35	0.493 ±0.268	3.023 ±0.423	0.0000
Wild Cordyceps V.S. *Cordyceps sinensis*	Sa (d16:0)	1.41	0.013 ±0.013	12.732 ±0.724	0.0000
	HexCer (d19:2/16:0(OH))	1.40	0.958 ±0.289	10.203 ±1.147	0.0000
	PI-Cer (d18:0/16:0(OH))	1.40	0	6.977 ±1.505	0.0000
Wild Cordyceps V.S. *Cephalospovium sinensis*	SM (d18:2/16:0(OH))	1.46	0.419 ±0.058	0.017 ±0.031	0.0000
	Cer (d18:1/18:1)	1.45	0.074 ±0.069	12.008 ±2.387	0.0000
	Cer (d18:0/18:0)	1.40	0.008 ±0.006	9.885 ±3.149	0.0000
Wild Cordyceps V.S. *Mortierella SP*	Cer (d18:0/16:1)	1.41	0.031 ±0.025	0.616 ±0.110	0.0000
	SM (d18:2/18:0)	1.40	10.426 ±2.543	0.145 ±0.051	0.0000
	SM (d18:2/16:0(OH))	1.37	0.419 ±0.058	0.054 ±0.016	0.0000
Wild Cordyceps V.S. *Gliocadium roseum*	Cer (d18:1/18:1)	1.45	0.074 ±0.069	13.455 ±0.594	0.0000
	Sa (d16:0)	1.44	0.013 ±0.013	0.211 ±0.005	0.0000
	Sa (m18:0)	1.44	0.046 ±0.011	0.283 ±0.024	0.0002
Hirsutella sinensis V.S. *Cordyceps sinensis*	Sa (d16:0)	1.28	0.014 ±0.006	12.732 ±0.724	0.0000
	So (t22:2)	1.28	0.252 ±0.071	10.170 ±0.732	0.0000
	So (d20:1)	1.27	2.329 ±0.583	12.913 ±0.829	0.0000
Hirsutella sinensis V.S. *Cephalospovium sinensis*	Cer (d18:1/16:1)	1.42	12.510 ±1.138	1.109 ±0.309	0.0000
	HexCer (d19:2/16:0(OH))	1.37	5.436 ±0.638	0.547 ±0.802	0.0000
	Cer (d18:1/18:1)	1.36	1.227 ±0.845	12.008 ±2.387	0.0000
Hirsutella sinensis V.S. *Mortierella SP*	HexCer (t19:1/16:1)	1.41	4.416 ±0.619	0.056 ±0.018	0.0000
	Cer (d18:2/18:1)	1.39	0.009 ±0.005	0.543 ±0.083	0.0000
	SM (d14:1/20:0)	1.35	11.618 ±2.342	1.080 ±0.410	0.0000
Hirsutella sinensis V.S. *Gliocadium roseum*	Sa (d16:0)	1.41	0.014 ±0.006	0.211 ±0.005	0.0000
	Sa (t18:0)	1.41	0.107 ±0.049	4.544 ±0.177	0.0000
	Cer (d18:1/18:1)	1.41	1.227 ±0.845	13.455 ±0.594	0.0000
Cordyceps sinensis V.S. *Cephalospovium sinensis*	Sa (d16:0)	1.56	12.732 ±0.724	0.33 ±0.174	0.0000
	Cer (d19:2/16:0(OH))	1.56	10.203 ±1.147	0.547 ±0.802	0.0000
	Cer (d18:1/16:1)	1.55	11.865 ±1.381	1.109 ±0.309	0.0000
Cordyceps sinensis V.S. *Mortierella SP*	Sa (d16:0)	1.43	12.732 ±0.724	0.068 ±0.033	0.0000
	Cer (d19:2/16:0(OH))	1.41	10.203 ±1.147	3.820 ±2.451	0.0001
	Cer (d18:2/18:1)	1.38	0.118 ±0.035	0.543 ±0.083	0.0000
Cordyceps sinensis V.S. *Gliocadium roseum*	Cer (d18:1/18:1)	1.28	0.446 ±0.396	13.455 ±0.594	0.0000
	Sa (t18:0)	1.28	0.674 ±0.121	4.544 ±0.177	0.0000
	Sa (t18:0) isomer	1.28	2.373 ±0.210	10.881 ±0.455	0.0000
Cephalospovium sinensis V.S. *Mortierella SP*	Cer (d18:2/16:1)	1.76	1.008 ±0.299	2.350 ±0.452	0.0000
	Cer (d18:1/18:1)	1.74	12.008 ±2.387	3.823 ±1.802	0.0000
	So (d16:1)	1.70	0.375 ±0.223	1.317 ±0.340	0.0000

Cephalospovium sinensis V.S. *Gliocadium roseum*	Sa (t18:0)	1.90	0.261 ±0.121	4.544 ±0.177	0.0000
	So (t18:1)	1.88	0.107 ±0.062	0.929 ±0.065	0.0000
	Cer (d18:1/14:1)	1.81	0.053 ±0.016	0.141 ±0.004	0.0000
Mortierella SP V.S. *Gliocadium roseum*	Sa (t18:0)	1.41	0.392 ±0.096	4.544 ±0.177	0.0000
	So (t18:1)	1.39	0.196 ±0.078	0.929 ±0.065	0.0000
	Cer (d18:2/18:1)	1.36	0.543 ±0.083	0.029 ±0.018	0.0000

Figure S1. Linear correlation of the spiked amounts with determined amounts of SPL standards.

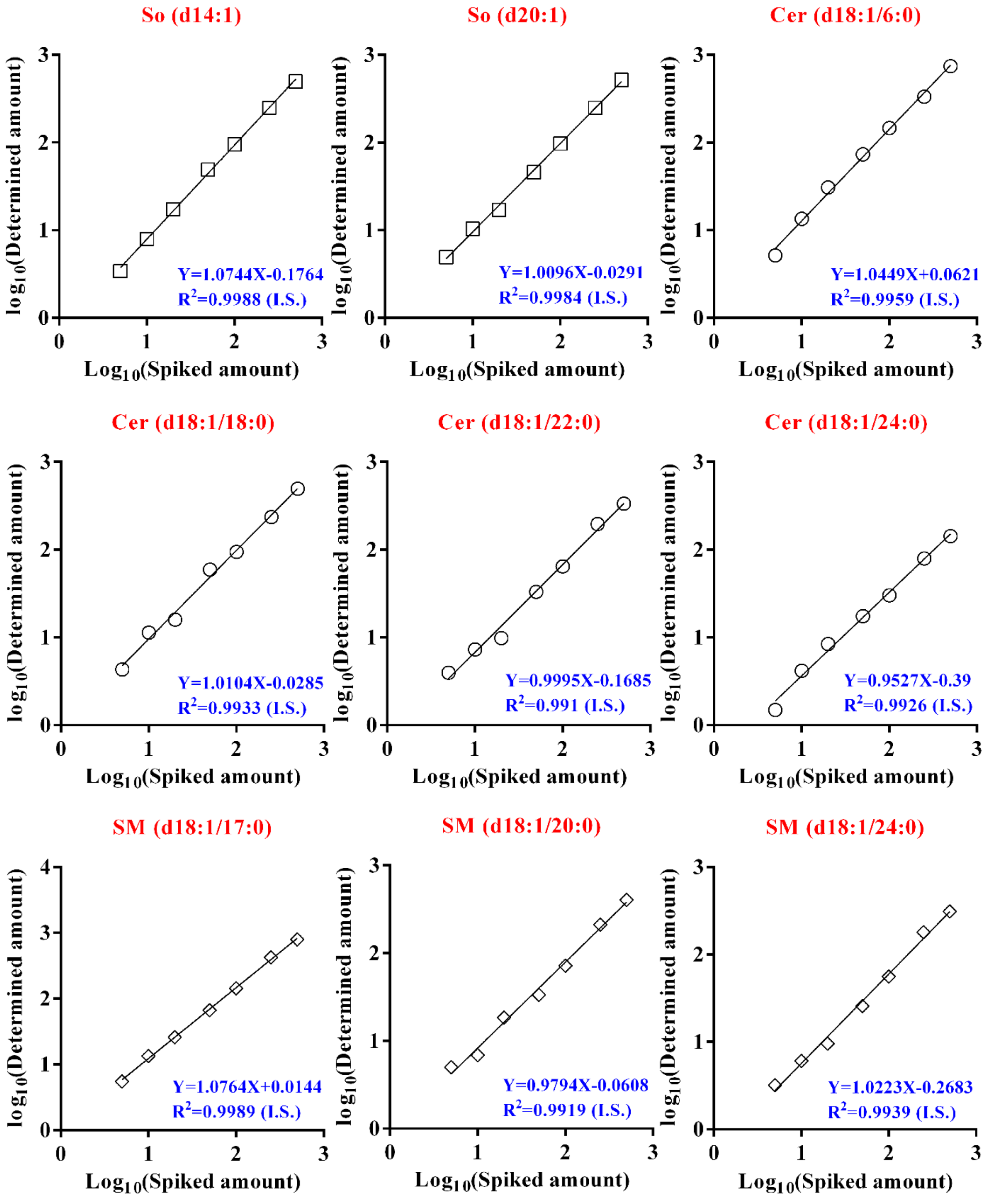

Figure S2. The total levels of four subclasses of ceramides (A, each bar represents mean ± SD) and the levels of 51 ceramides (B, each bar represents mean value of individual ceramides) in wild Cordyceps and its mycelia.

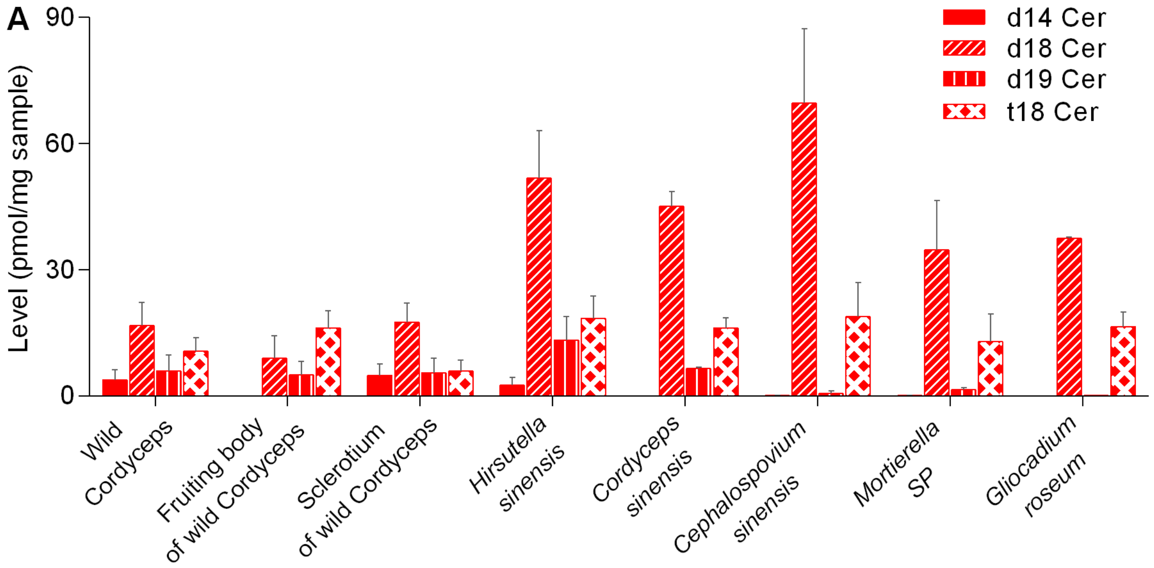

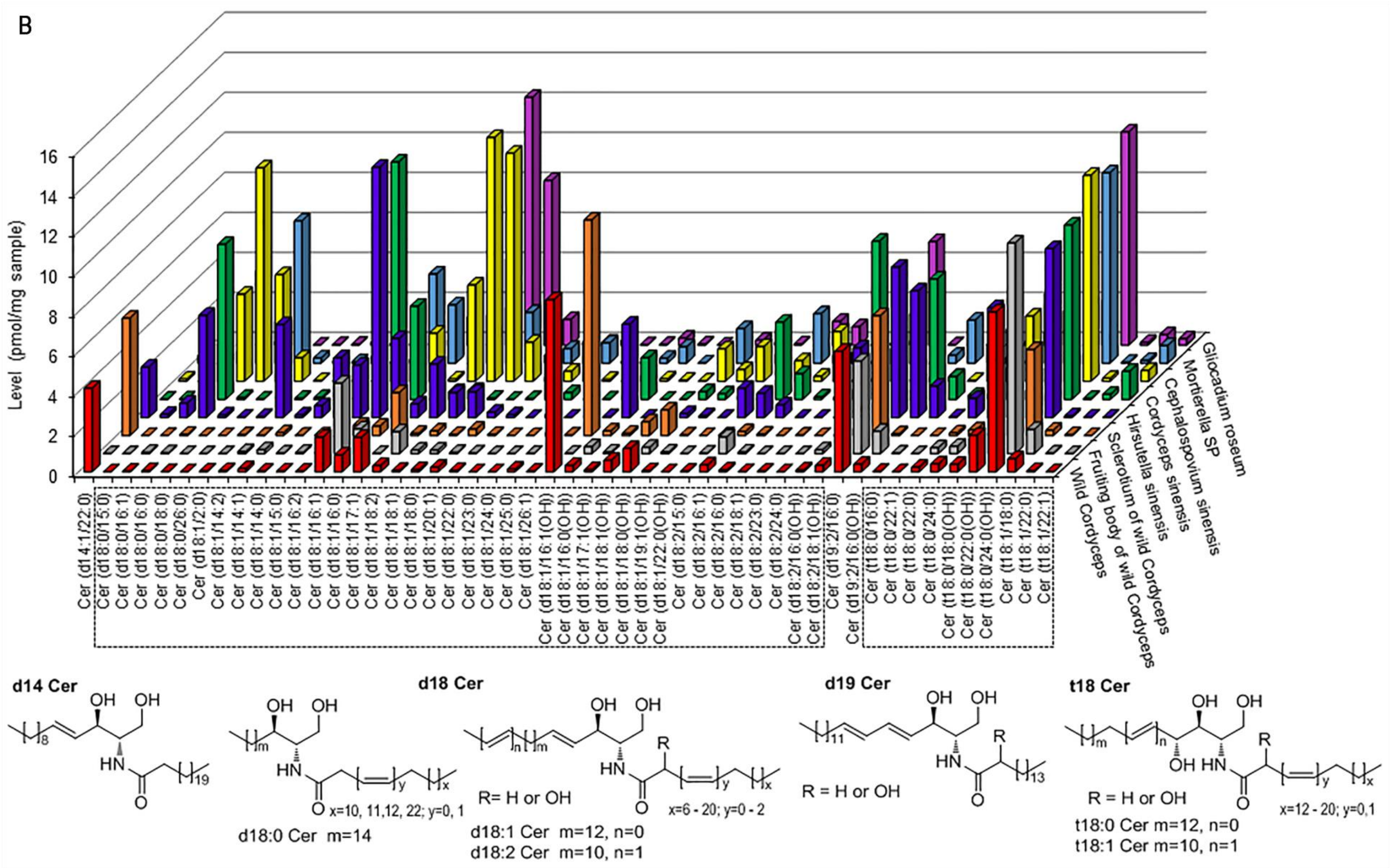

B
Level (pmol/mg sample)
0
2
4
6
8
10
12
14
16
Wild Cordyceps
Fruiting body of wild Cordyceps
Sclerotium of wild Cordyceps
Hirsutella sinensis
Cordyceps sinensis
Cephalospovium sinensis
Mortierella SP
Gliocadium roseum
d14 Cer
d18 Cer
d19 Cer
t18 Cer
R= H or OH
R = H or OH
x=10, 11,12, 22; y=0, 1
d18:0 Cer m=14
x=6 - 20; y=0 - 2
d18:1 Cer m=12, n=0
d18:2 Cer m=10, n=1
x=12 - 20; y=0,1
t18:0 Cer m=12, n=0
t18:1 Cer m=10, n=1

Figure S3. The total levels of glycosphingolipids (A, each bar represents mean ± SD) and the levels of 8 glycosphingolipids (B, each bar represents mean value of individual glycosphingolipids) in wild Cordyceps and its mycelia.

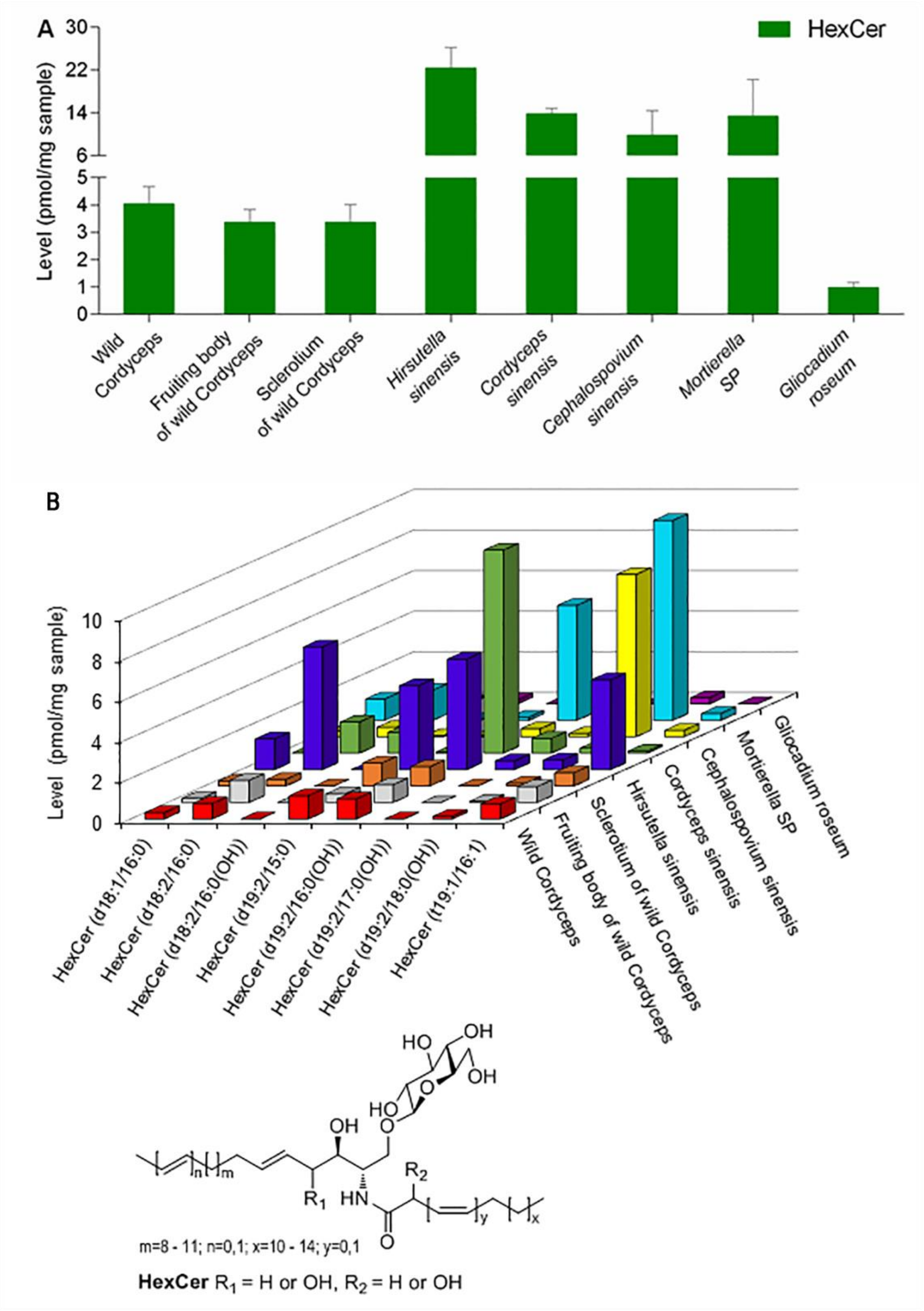

Supplement: Supplementary Information [file srep20870-s1.pdf]
